# *Staphylococcus aureus* exhibits spatiotemporal heterogeneity in Sae activity during kidney abscess development

**DOI:** 10.1128/mbio.02043-25

**Published:** 2025-11-13

**Authors:** Anjali Anil, Rezia Era D. Braza, Bessie Liu, Valerie Altouma, Clement Adedeji, Ananyaa Welling, Alysha L. Ellison, Megan H. Check, Irnov Irnov, Rachel Prescott, Victor J. Torres, Kimberly M. Davis

**Affiliations:** 1 W. Harry Feinstone Department of Molecular Microbiology and Immunology, Johns Hopkins Bloomberg School of Public Health 25802 , Baltimore, Maryland, USA; 2 Department of Microbiology, New York University Grossman School of Medicine 12296, New York, New York, USA; 3 Department of Host-Microbe Interactions, St. Jude Children’s Research Hospital 5417, Memphis, Tennessee, USA; University of Illinois Chicago, Chicago, Illinois, USA

**Keywords:** *Staphylococcus aureus*, virulence factors, heterogeneity, kidney abscess

## Abstract

Virulence factors are required for bacterial pathogens to establish infection; however, their expression can be energetically costly and must be tightly controlled to avoid fitness costs. Expression can be controlled at specific stages during infection (temporal regulation) or expressed by small subsets of the bacterial population (spatial regulation). There has been a great deal of interest in developing virulence factor-targeting strategies to combat *Staphylococcus aureus* infection, but the spatiotemporal regulation of the virulence factor master regulatory systems (Agr, Sae) has not been explored during kidney abscess formation. This information is critical for the design of therapeutics targeting these pathways. Here, we utilized a fluorescent transcriptional reporter approach to visualize dynamics in Agr and Sae activity during abscess formation in the mouse kidney. We categorized kidney abscess formation into four stages, then defined spatiotemporal gene expression. Agr signaling appeared inactive in the kidney; consistent with this, *agr* mutant abscesses fully developed. In contrast, we observed heterogeneous (ON/OFF) activity of Sae at early stages, where bacteria were found intracellularly within neutrophils. Sae activity increased as abscesses developed, and heterogeneity in spatial patterning was observed, but patterns varied between abscesses, suggesting distinct microenvironments within individual abscesses. Consistent with a requirement for Sae activity during abscess development, the *sae* mutant did not develop abscesses past early stages. These results have implications for the genes regulated by Agr and Sae and suggest a requirement for Sae activity during kidney abscess development.

**IMPORTANCE:**

Infections with *Staphylococcus aureus* pose a serious public health threat due to high levels of antibiotic resistance and limited efficacy of alternative therapeutics. There has been a great deal of interest in developing novel therapeutics that target virulence factors essential during infection. However, it remains largely unknown if these factors are required at specific stages of the infection, and whether all bacterial cells or a limited subset express them. Here, we sought to examine virulence factor expression using fluorescent reporter strains that would indicate activity of two master regulators of virulence in *S. aureus*, Agr and Sae. While Agr appeared inactive during kidney abscess development, the Sae system exhibited heterogeneity, increased expression at later stages, and was required for abscess progression. These results provide critical information for the development of virulence factor-targeting strategies for kidney abscess treatment.

## INTRODUCTION

Methicillin-resistant *Staphylococcus aureus* (MRSA) infections are a major cause of morbidity and mortality worldwide (1, 2), and effective therapies are scarce (3). Failure of vaccine or drug candidates in clinical trials has been largely attributed to the pathogen’s diverse array of virulence factors that subvert the host immune response (4). Neutralizing the effects of key virulence factors could curtail infection progression, making them attractive therapeutic targets (5). However, virulence factor expression can be energetically costly, and bacteria have adapted to express these gene products only at specific sites and stages of infection or within certain subsets of bacteria to minimize their fitness costs (6).

A hallmark of *S. aureus* systemic infection in mice is the formation of kidney abscesses. Abscesses consist of a central bacterial core, referred to as a staphylococcal abscess community (SAC), surrounded by necrotic and viable immune cells (7). Abscess formation sequesters bacteria, limiting further spread, yet clearance of the SAC by recruited immune cells is often unsuccessful, and treatment requires antibiotic therapy, surgical intervention, or both (8).

Previous studies have shown that a diverse array of virulence factors is necessary for abscess formation (9). Many of these factors are regulated by the accessory gene regulatory system (Agr) and the *S. aureus* exoprotein expression (Sae) two-component systems (10, 11). Agr is a quorum-sensing system comprised of four genes (*agrB, agrD, agrC, agrA*) that modulates *S. aureus* gene expression as a function of its population density (12, 13). The *agrD-*encoded autoinducing peptide is processed by AgrB and sensed by the AgrC histidine kinase, resulting in AgrA-dependent gene expression. Agr-regulated virulence factors include phenol-soluble modulins, alpha-toxin, and certain toxins that are cytotoxic to immune cells (leukocidins). The SaeS histidine kinase is activated by immune cell components (human alpha-defensins, reactive oxygen species), nutrient limitation, or membrane perturbation (14–16), promoting SaeR-dependent expression of leukocidins and adhesins (9, 17). Agr contributes to abscess formation in the skin (5), but the direct contribution of Agr to kidney abscess formation has not been examined. Sae-regulated virulence factors promote kidney abscess development (9), but whether Sae activity is needed at a particular stage or within a particular bacterial subpopulation is not known. The spatiotemporal expression patterns of *S. aureus* virulence factors are an understudied component of abscess pathogenesis that could help inform new therapeutic approaches.

In this study, we utilized fluorescent reporter strains in the CA-MRSA USA300 LAC background in a mouse model of infection to define spatiotemporal changes in the activities of Agr and Sae during kidney abscess development. The *agr* P2 promoter and the inducible *sae* P1 promoter (15, 18) were fused to *gfp* in distinct fluorescent reporter strains. We found the Agr system was inactive during kidney abscess development, while Sae activity was high within mature SACs. We also observed significant heterogeneity in Sae activity between bacterial subpopulations at different stages of infection. Using *agr* and *sae* mutant strains, we demonstrate that while Agr is dispensable, loss of Sae significantly impairs SAC development. Ultimately, our study underscores the significance of considering niche and stage specificity of virulence factor expression, and the presence of non-expressing bacterial subsets, in the development of virulence factor-targeting therapeutics.

## RESULTS

### Retro-orbital inoculation results in gradual expansion of the *S. aureus* population in the mouse kidney

We hypothesized that *S. aureus* would exhibit spatiotemporal regulation of virulence factor expression during kidney abscess formation; to test this hypothesis, we generated a series of fluorescent reporter strains. All strains contain a *P_sarA_::sod* RBS::*mCherry* cassette integrated at the SapI1 attachment site for constitutive expression of mCherry (19, 20). The mCherry signal is an internal control for transcriptional activity of individual cells and allows us to quantify relative reporter expression normalized to mCherry. Transcriptional reporter constructs consist of the P2 promoter of the *agr* operon (*P_agrB_::gfp*) or the P1 promoter of the *sae* operon (*P_saeP_::gfp*) upstream of a *sod* ribosomal binding site (to ensure robust translation of the transcripts) fused to superfolder-GFP (*gfp*). The GFP negative control strain (GFP^−^) has an insertion of a promoter-less version of *gfp*, serving as both a baseline control for promoter-independent *gfp* expression and detection of cellular autofluorescence. All *gfp* constructs were integrated into a neutral site in the mCherry^+^ strain genome (21).

**Fig 1:**
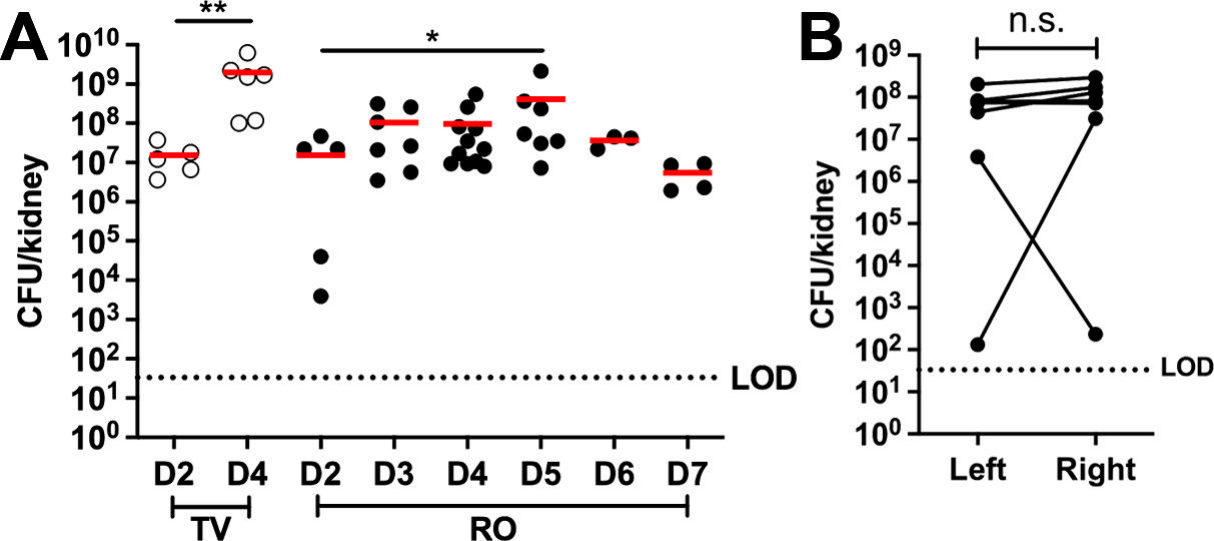
Retro-orbital inoculation results in gradual expansion of the *S. aureus* population in the mouse kidney. C57BL/6 mice were intravenously inoculated with 10^6^ CFU of the *S. aureus* GFP^−^ control strain (constitutive mCherry: *P_sarA_::sod* RBS::*mCherry*) via the TV or RO route. (**A**) Bacterial load (CFU/kidney) at the indicated time points (D = day). Each dot represents one mouse. *N* = 3 to 11 mice per time point. Red bars: mean. (**B**) Comparison of bacterial load in the right and left kidneys at D4 after RO inoculation. *N* = 7 mice. Lines connect pairs of kidneys from the same mouse. Horizontal dotted line: limit of detection (LOD). Statistics: (**A**) Mann-Whitney *t*-test; (**B**) Wilcoxon matched-pairs test. **P < 0.01, *P < 0.05, n.s.: not significant.

We performed initial experiments with the GFP^−^ control strain employing a mouse model of systemic spread-derived kidney infection (22). We compared retro-orbital (RO) and tail vein (TV) inoculations, two commonly used routes for *S. aureus* systemic administration in mice (9, 23), given recent reports that the two intravenous routes can differ in pathogen delivery (24). TV inoculation with the GFP^−^ control strain resulted in significantly higher bacterial load in the kidney at day 4 vs day 2 post-inoculation (p.i.) (Fig. 1A). The relatively high bacterial load at day 2 resulted in high levels of early tissue damage, early signs of morbidity, and suggested bacteria trafficked rapidly from the liver to the kidney instead of experiencing a bottleneck (23). For mice infected retro-orbitally (RO), kidney CFUs increased between days 2 and 5 p.i., suggesting initial bacterial seeding and population expansion within this timeframe, beyond which a gradual decline suggests some infection control (Fig. 1A). For all subsequent experiments, we employed the RO route to capture kidney abscess dynamics (seeding, expansion). We also compared the bacterial load in the left and right kidneys of RO-inoculated mice at day 4 p.i. and observed no significant difference (Fig. 1B). However, two of seven mice had a lower bacterial load and a >10^4^-fold difference in CFUs between the two kidneys. For consistency, the left kidney was used for CFU enumeration, and the right kidney was used for histology in subsequent experiments.

### Defining the four observed stages of *S. aureus* mouse kidney abscess development

**Fig 2:**
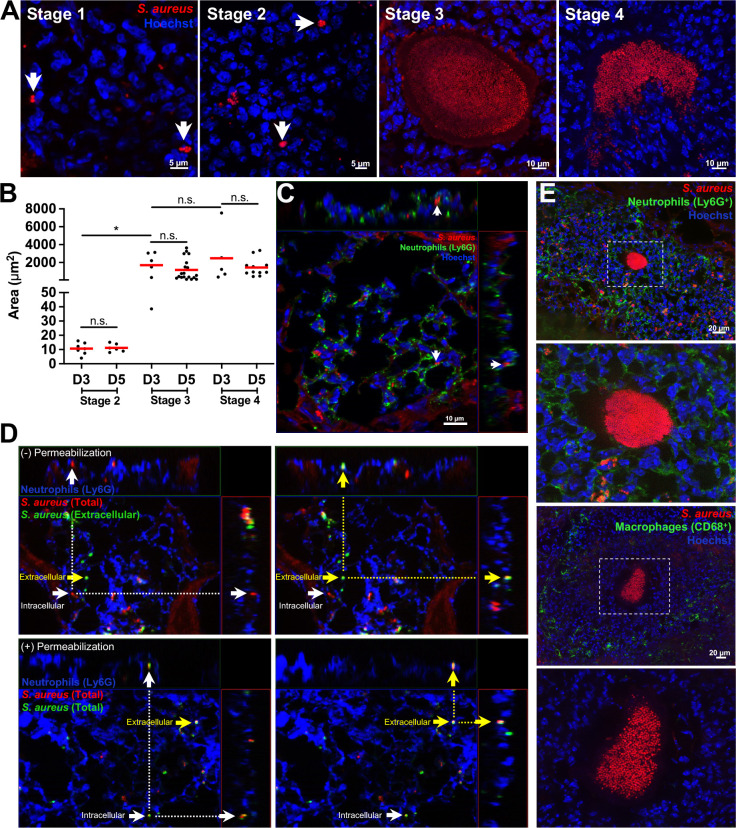
Defining the four observed stages of *S. aureus* mouse kidney abscess development. C57BL/6 mice were inoculated RO with the *S. aureus* GFP^−^ control strain. At the indicated time points (day 3 [D3] to day 5 [D5]), mice were sacrificed, and kidneys were harvested. (**A**) Representative images depicting stages of S. *aureus* kidney abscess development. Stage 1: intracellular *S. aureus* (white arrow), stage 2: small extracellular clusters (white arrow), stage 3: fully formed and intact SAC, and stage 4: dispersed SAC. (**B**) Areas of *S. aureus* extracellular clusters (stage 2) or SACs (stages 3 and 4) at D3 and D5. Dots represent individual extracellular clusters (stage 2) or SACs (stages 3 and 4). *N* = 3 to 4 mice per time point. Red bars: mean. (**C**) Immunofluorescence images showing *S. aureus* inside neutrophils (Ly6G^+^). XZ and YZ planes corresponding to the point highlighted by the white arrow are shown at the top and right, respectively. (**D**) Immunofluorescence microscopy differentiating extracellular vs intracellular mCherry^+^ *S. aureus* (within Ly6G^+^ neutrophils) by staining with antisera (green) either before (top row) or after (bottom row) permeabilization. Extracellular bacteria were stained with antisera prior to permeabilization, while intracellular bacteria were only accessible to antisera after permeabilization. Dotted lines connect objects in the XZ and YZ planes. (**E**) Representative immunofluorescence images showing localization of neutrophils (Ly6G^+^, top row) and macrophages (CD68^+^, bottom row) around a stage 3 SAC. Right column: zoom of the white boxed area. Statistics: (**C**) Kruskal-Wallis one-way analysis of variance (ANOVA) with Dunn’s post-test. *P < 0.05, n.s.: not significant.

We next sought to visualize and define the stages of *S. aureus* strain LAC kidney abscess development by fluorescence microscopy using the published framework with *S. aureus* strain Newman as a guide (9). C57BL/6 mice were inoculated RO with the GFP^−^ control strain, kidneys were harvested on days 3 and 5 p.i., and tissue was prepared for immunofluorescence microscopy. Analysis of kidney sections revealed distinct stages of abscess development, which we categorized into four stages, with both similarities and differences relative to previous studies (9) (Fig. 2A and B). Stage 1 was defined by the presence of intracellular *S. aureus* within neutrophils, based on Ly6G^+^ neutrophil membrane signal surrounding bacteria (Fig. 2C) and membrane protection from *S. aureus* antisera staining prior to permeabilization (Fig. 2D). This was consistent with Pollitt et al. demonstrating neutrophils promote dissemination of *S. aureus* into the kidney (23), but this definition is distinct from the bloodstream stage 1 designation in Cheng et al. (25). Stage 2 was defined as small extracellular clusters of *S. aureus*, ranging in area between 4.03 to 16.07 µm^2^ (Fig. 2A and B). A fully formed, intact SAC was categorized as stage 3, similar to previous designations (25). Most stage 3 SACs had a space (frequently autofluorescent) between bacterial cells and the surrounding host cells, pointing to the presence of the fibrin pseudocapsule and microcolony-associated meshwork (17, 26). The area of stage 3 SACs ranged from 38.49 to 3,129.52 µm^2^, with many SACs in the 100–1,000 µm^2^ range (Fig. 2B). We observed some SACs were disrupted, with bacterial-host cell interactions at multiple points on the SAC; this was classified as stage 4 (Fig. 2A). Many stage 4 abscesses also had host cells interacting with small clusters of *S. aureus* nearby, suggesting phagocytic uptake of bacteria that were previously part of the SAC (Fig. S1A). Multiple stages could be observed within a single kidney across time points, but the proportion of stages shifted over time (day 3 vs day 5).

Immunofluorescence microscopy was used to visualize the localization of neutrophils (Ly6G^+^) and macrophages (CD68^+^) relative to *S. aureus*. We observed neutrophils near *S. aureus* at each stage, and stage 3 and 4 SACs were surrounded by large numbers of neutrophils (Fig. 2E, top two panels). Based on previous studies, this represents a mixture of non-viable and viable neutrophils (9). Although macrophages could be found near stage 1 and 2 bacteria, they were present at much lower numbers than neutrophils (Fig. S1B). A characteristic macrophage ring was observed around stage 3 and 4 abscesses outside the neutrophil layer, away from the SAC (Fig. 2E, bottom two panels) (27). This indicates that while macrophages are present within abscesses, *S. aureus* is predominantly interacting with neutrophils.

### Characterizing Agr and Sae fluorescent reporter activity in culture

Before using fluorescent reporter strains in infections, we confirmed that the *agr* and *sae* reporter strains and GFP^−^ control exhibit similar growth kinetics in liquid media (Fig. S2A). The *agr* reporter strain (*P_agrB_::gfp*) demonstrated a growth-dependent increase in reporter expression (GFP/mCherry ratio) (Fig. S2B) (13), while the *sae* reporter strain (*P_saeP_::gfp*) showed low expression in the exponential phase, as expected (Fig. S2B) (28). It is important to note that this GFP variant is quite stable in exponential-phase cells (Fig. S2C), which means fluorescence could be detected after transcript levels decrease. As expected, GFP^−^ control colonies had very low GFP fluorescence, while *agr* and *sae* reporter colonies had high and intermediate GFP fluorescence, respectively (Fig. S2D). Flow cytometric analysis indicated >90% of cells are *agr-*expressing in the stationary phase (8 vs 24 h culture), whereas *sae*-expressing cells increase in percentage and mean fluorescence intensity between 8 and 24 h culture (Fig. S2E through G). Reporter strain transcript levels of *agrB* or *saeP* closely mirror *gfp*; however, discordance relative to signal could be due to fluorescent protein stability (Fig. S2C and H).

### *agr* reporter expression is not above baseline during kidney abscess development

To understand how Agr and Sae activity change during abscess development, we infected mice with the GFP^−^ control, *agr* (*P_agrB_::gfp*), or *sae* (*P_saeP_::gfp*) reporter strains. Kidneys were harvested at days 3, 4, and 5 p.i., corresponding with periods of peak bacterial growth within the kidneys. We did not detect significant differences in bacterial load, except for a slight decrease in *sae* reporter CFUs at day 5 p.i. (Fig. S3A).

To quantify reporter expression, the constitutive mCherry signal was used to identify bacterial cells, and the total GFP (reporter) and mCherry signals were expressed as a GFP/mCherry ratio. The number of events per stage analyzed for each mouse is summarized in Table S1. The GFP/mCherry ratio of *S. aureus* GFP^−^ control cells revealed inter- and intra-stage heterogeneity across time points (Fig. S3B). This could arise from leaky *gfp* expression, differences in tissue autofluorescence, or differences in mCherry expression due to changes in bacterial metabolic state. To account for this, the GFP/mCherry ratio of each event was normalized to the average GFP^−^ control value at the same stage and day (black dashed baseline at Y = 1) (Fig. S3C). A comparison of *agr* and *sae* reporter strain values before and after normalization to the GFP^−^ control strain is shown in Fig. S3D through G.

**Fig 3:**
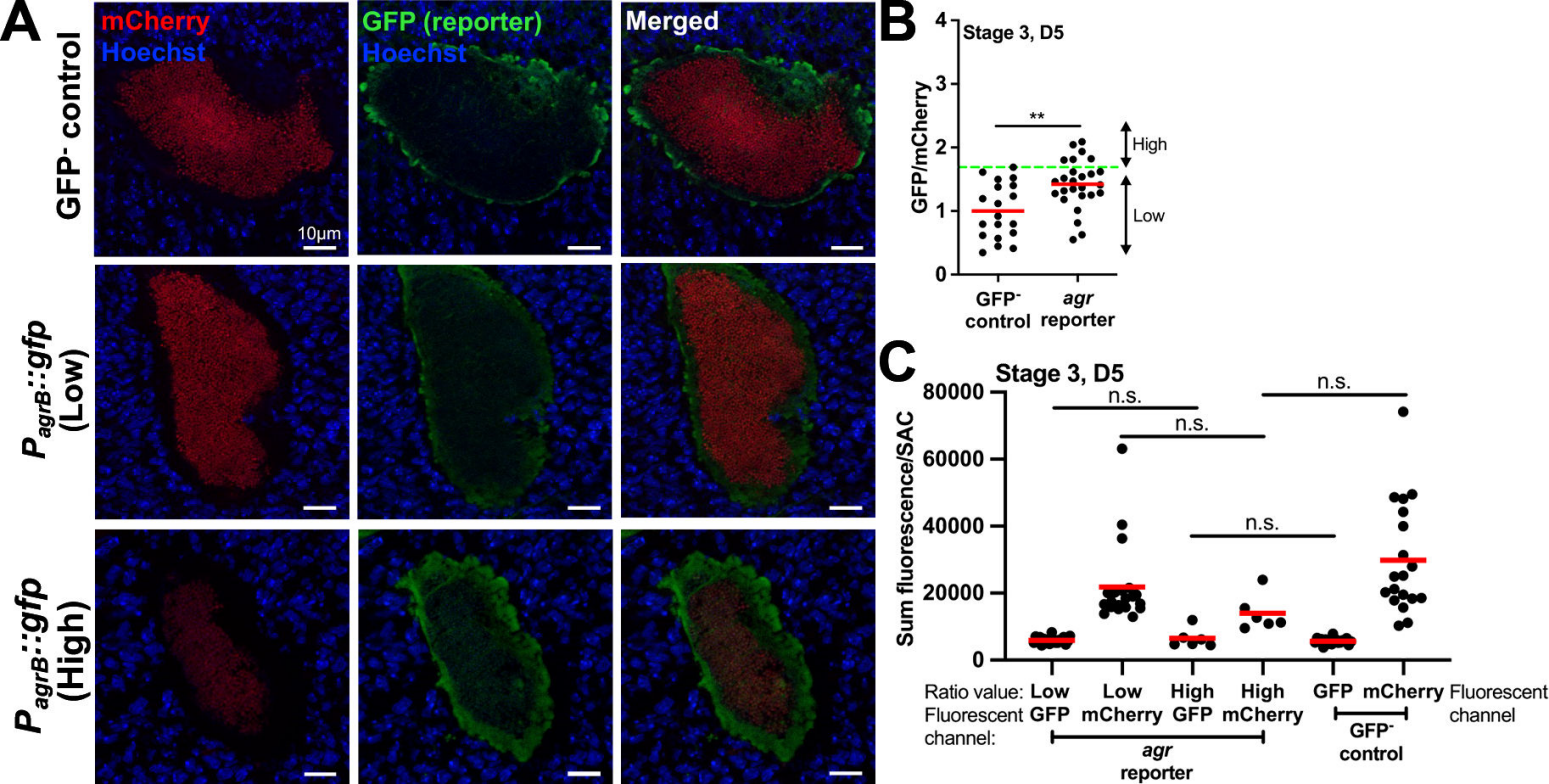
*agr* reporter expression is not above baseline during kidney abscess development. C57BL/6 mice were inoculated with GFP^−^ control or *agr* reporter (*P_agrB_::gfp*) strains, both expressing mCherry constitutively. Mice were sacrificed at day 5, and the kidneys were harvested and processed for fluorescence microscopy. (**A**) Representative images for data shown in panel B. (**B**) Comparison of fluorescent signals in GFP^−^ control and *agr* reporter stage 3 SACs at day 5 p.i. Green dashed horizontal line: highest GFP/mCherry value for the GFP^−^ control, used to differentiate reporter High and Low SACs for the *agr* reporter. (**C**) Sum fluorescence/SAC of individual fluorescent channels (GFP, mCherry). *agr* reporter data sets are split based on the Low or High ratio designation in panel B. All SACs from panel B are analyzed in panel C. Dots: individual SACs. *N* = 3 to 4 mice per time point. Red bars: mean. Statistics: (**B**) Mann-Whitney; (**C**) Kruskal-Wallis one-way ANOVA with Dunn’s post-test; **P < 0.01, n.s.: not significant.

We first analyzed *agr* reporter expression within intracellular *S. aureus* (stage 1) and found the average GFP/mCherry ratios were below the GFP^−^ control baseline, indicating the Agr system is off (Fig. S3E). In stages 2–4, the *agr* GFP/mCherry ratio was statistically above baseline only in intact SACs (stage 3) at day 5 p.i. (Fig. S3E). Within these SACs, most had GFP/mCherry ratios similar to the GFP^−^ control (Low), while a few displayed values above baseline (High) (Fig. 3A and B). We also observed decreased mCherry signal (Fig. 3A) in some SACs, which could lead to increased ratios. We then analyzed the signals separately and found no significant changes in GFP or mCherry signals across groups (Fig. 3C), indicating *agr* expression was not above baseline during kidney abscess development.

### *sae* reporter expression is highly heterogeneous in intracellular *S. aureus* but is more uniformly high within intact abscesses

**Fig 4:**
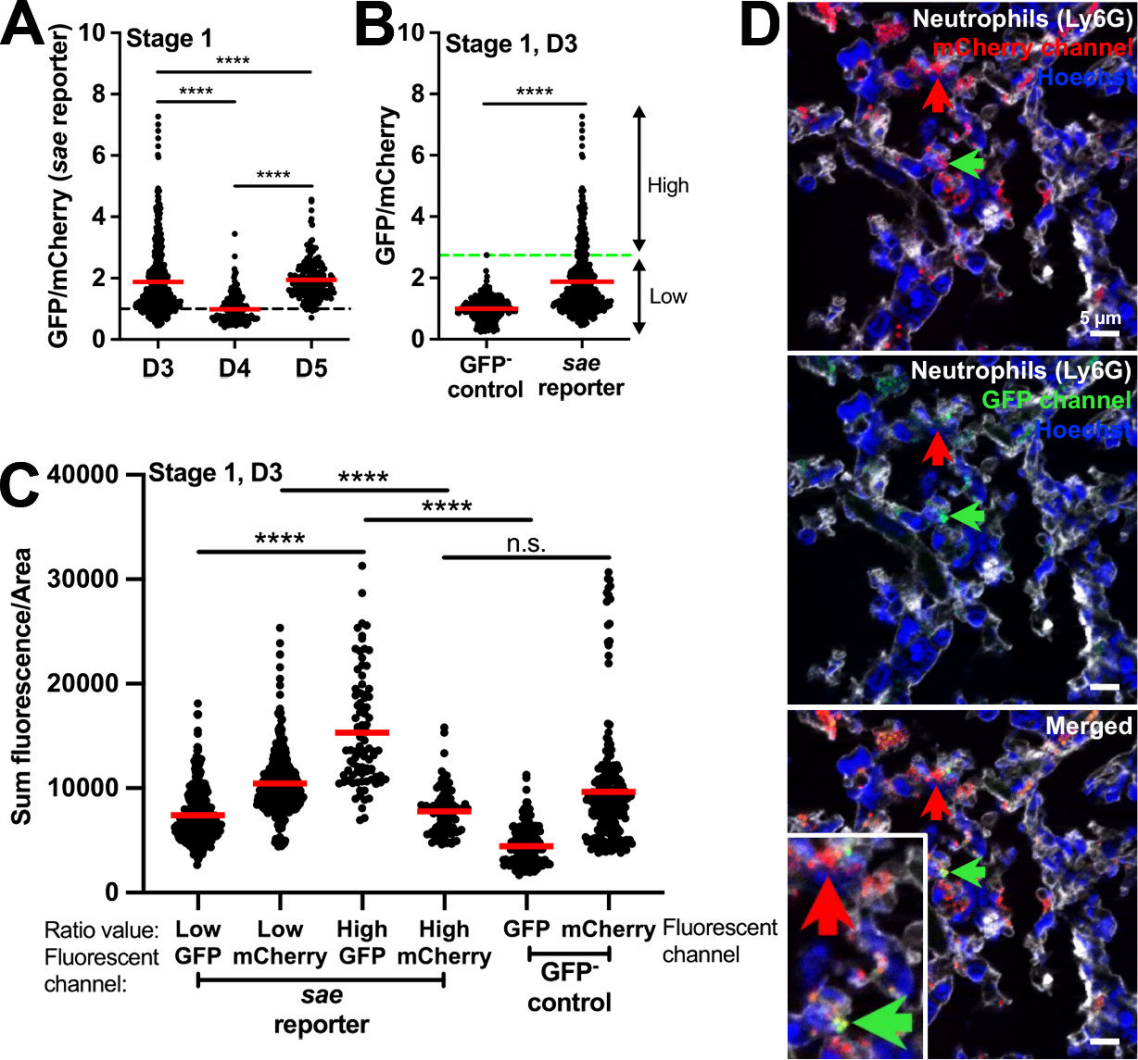
*sae* reporter expression is highly heterogeneous in intracellular *S. aureus*. C57BL/6 mice were inoculated with GFP^−^ control or *sae* reporter (*P_saeP_::gfp*) strains, both expressing mCherry constitutively. Mice were sacrificed at days 3, 4, or 5, and kidneys were harvested and processed for fluorescence microscopy. The GFP/mCherry ratio of each *sae* reporter event was normalized to the average value of GFP/mCherry ratios of the respective stage and day-matched GFP^−^ control events (represented by the black dotted baseline at Y = 1 in panels A and B). (**A**) *sae* reporter expression in stage 1 *S*. *aureus*. Dots represent intracellular events. (**B**) Comparison of fluorescent signals in GFP^−^ control and *sae* reporter stage 1 events at day 3 p.i. Green dashed horizontal line: highest GFP/mCherry value for the GFP^−^ control, used to differentiate reporter Low and High events for the *sae* reporter. (**C**) Sum fluorescence/area of individual fluorescent channels (GFP, mCherry). *sae* reporter data sets are split based on the Low or High ratio designation in panel B. All SACs from panel B are analyzed in panel C. (**D**) Representative images for the data shown in panel B. Red arrow represents *sae* non-expressing (Low) *S. aureus*, green arrow represents *sae*-expressing (High). Inset in the merged image is a zoom-in of the highlighted Low and High cells. *N* = 3 to 5 mice per time point. Red bars: mean. Statistics: (**A**) and (**C**) Kruskal-Wallis one-way ANOVA with Dunn’s post-test; (**B**) Mann-Whitney ****P < 0.0001, n.s.: not significant.

**Fig 5:**
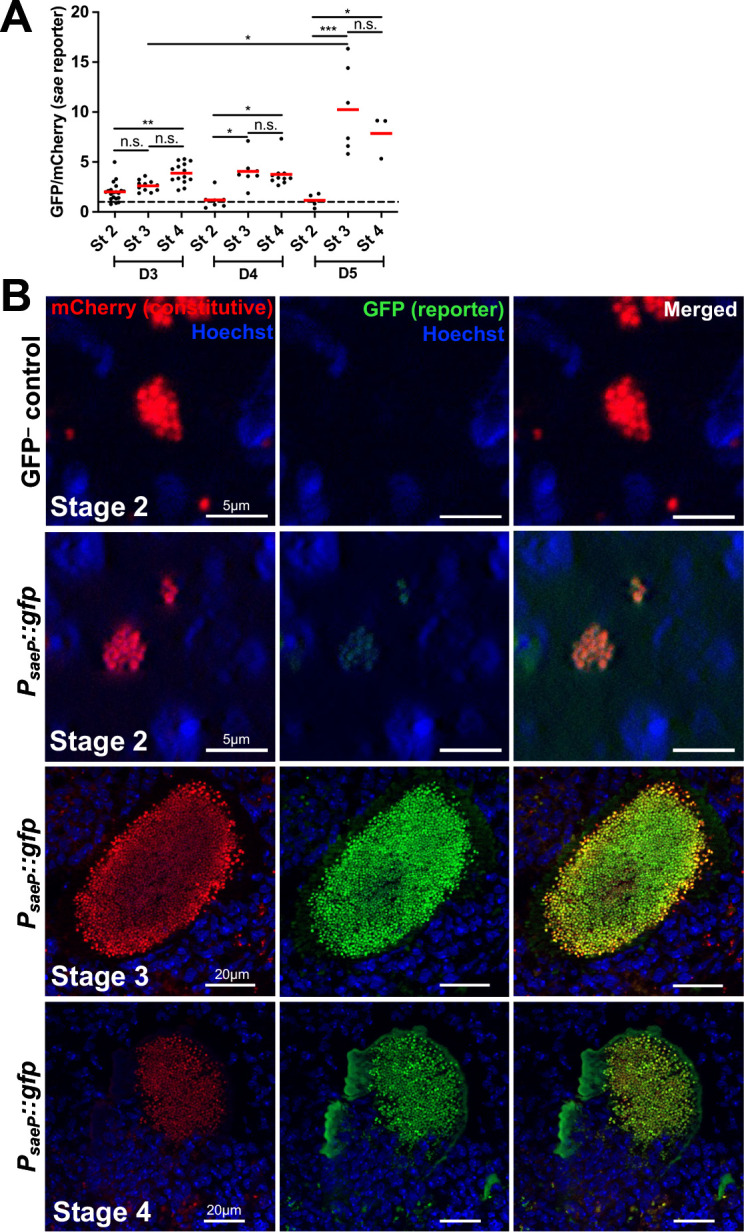
*sae* reporter expression is higher in fully formed SACs as compared to small extracellular clusters. C57BL/6 mice were inoculated with GFP^−^ control or *sae* reporter strains, both expressing mCherry constitutively. Mice were sacrificed at days 3, 4, or 5, and kidneys were harvested and processed for fluorescence microscopy. The GFP/mCherry ratio of each *sae* reporter event was normalized to the average value of GFP^−^ control events (represented by the black dotted baseline at Y = 1 in panel A). (**A**) *sae* reporter expression in abscess stages 2 to 4. Dots represent individual events. *N* = 3 to 5 mice per time point. Red bars: mean. (**B**) Representative images. Statistics: (**A**) Kruskal-Wallis one-way ANOVA with Dunn’s post-test. ***P < 0.001, **P < 0.01, *P < 0.05, n.s.: not significant.

We next analyzed *sae* reporter expression during abscess development. Unlike the *agr* reporter, average *sae* reporter values were higher than baseline at stage 1 on days 3 and 5 p.i., suggesting that the Sae system is generally active during intracellular residence of *S. aureus* (Fig. 4A). Interestingly, a subset of stage 1 *S*. *aureus* exhibited *sae* expression levels (GFP/mCherry) similar to the GFP^−^ control (Low), while other bacteria had higher expression levels (High, 18.7% of the population) (Fig. 4B). Analysis of individual fluorescent channels indicated that the GFP signal was significantly above baseline for “High” cells without a change in mCherry (compared to the GFP^−^ control), confirming these cells were *sae*-expressing (Fig. 4C). We also observed that neutrophils (Ly6G^+^) harbor both subsets of *sae*-Low and *sae-*High *S. aureus* (Fig. 4D). At stage 2, *sae* expression was only above baseline on day 3. *sae* signal was high within stages 3 and 4, indicating the Sae system is highly active within intact and dispersed SACs (Fig. 5A and B). It is important to note that GFP stability could contribute to the stage 4 signal (Fig. S2C and H).

### The *sae* reporter exhibits heterogeneity in spatial expression patterns across SACs

**Fig 6:**
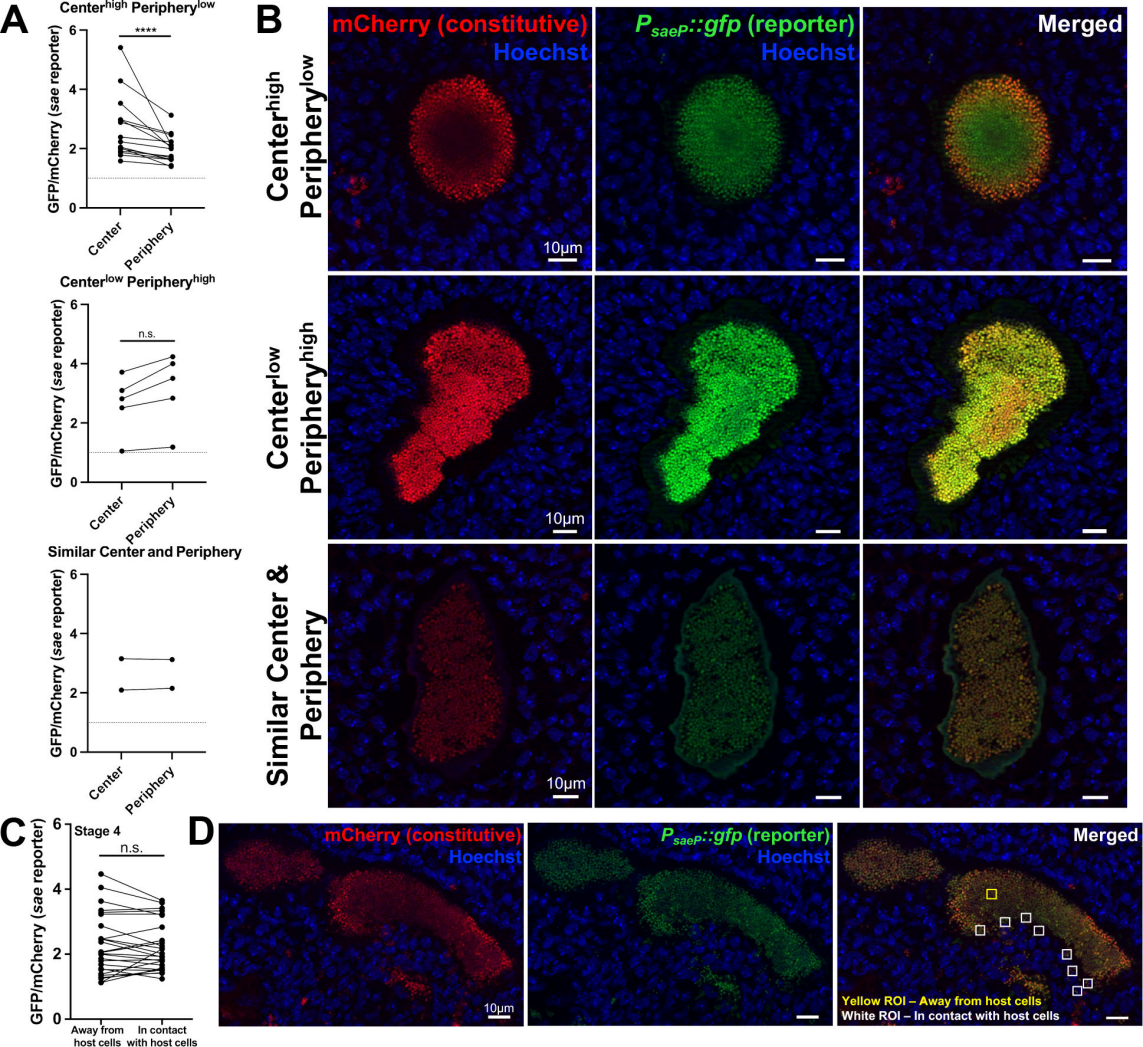
The *sae* reporter exhibits heterogeneity in spatial expression patterns across SACs. C57BL/6 mice were inoculated with the *sae* reporter strain. Mice were sacrificed at days 3, 4, or 5, kidneys were harvested and processed for fluorescence microscopy. Stage 3 (**A, B**): one region of interest (ROI, 4 µm^2^) at the center and eight ROIs along the periphery were selected. The GFP/mCherry ratio for each ROI was calculated using the sum GFP and sum mCherry values. The GFP/mCherry ratio of the center and the average GFP/mCherry value of the eight peripheral ROIs are shown. Stage 4 (panels **C, D**): one ROI in the center (away from host cells) and eight ROIs along the rupture (in contact with host cells) were selected. The GFP/mCherry ratio at the center vs averaged periphery of (**A**) stage 3, combined data from D3 to D5, split by spatial phenotype; and (**C**) stage 4, combined data from D3 to D5. Lines connect values from the same SAC. Representative images are shown in panels B and D. *N* = 3 to 5 mice per time point. Statistics: (**A**) and (**C**) Wilcoxon matched-pairs test. *P < 0.05, n.s.: not significant.

We hypothesized that peripheral bacteria within SACs (stages 3 and 4) may experience a different microenvironment than bacteria at the SAC center due to immune cell interactions and higher concentrations of nutrients. Since these signals could modulate Sae activity, we sought to determine if there were spatial differences in *sae* expression within individual SACs. We observed three phenotypes of SACs: 15/22 were Center^high^ Periphery^low^ (center to periphery > 1.0), 5/22 had lower center expression (Center^low^ Periphery^high^, center to periphery < 1.0), and 2/22 had similar expression at the center and periphery (center to periphery = 1.0) (Fig. 6A and B). At stage 4, we compared *sae* expression in peripheral *S. aureus* that were “in contact with host cells” (rupture point) with bacteria embedded deep within the SAC (“away from host cells”) (Fig. 6C and D). Here, we also observed three phenotypes: 11/26 with higher expression away from host cells (>1.0), 8/26 with lower expression away from host cells (<1.0), and 7/26 with equivalent expression at both locations (=1.0). However, differences were modest (range: 0.52–1.28) (Fig. 6C). The GFP and mCherry values at the center and periphery for each SAC are shown in Fig. S4A and B, and indicate that while the mCherry signal may seem heightened at the periphery of some SACs, this is not consistently the case. Collectively, these data indicate that *sae* reporter expression is highly heterogeneous between individual abscesses.

### Loss of *sae* but not *agr* impacts abscess development

**Fig 7:**
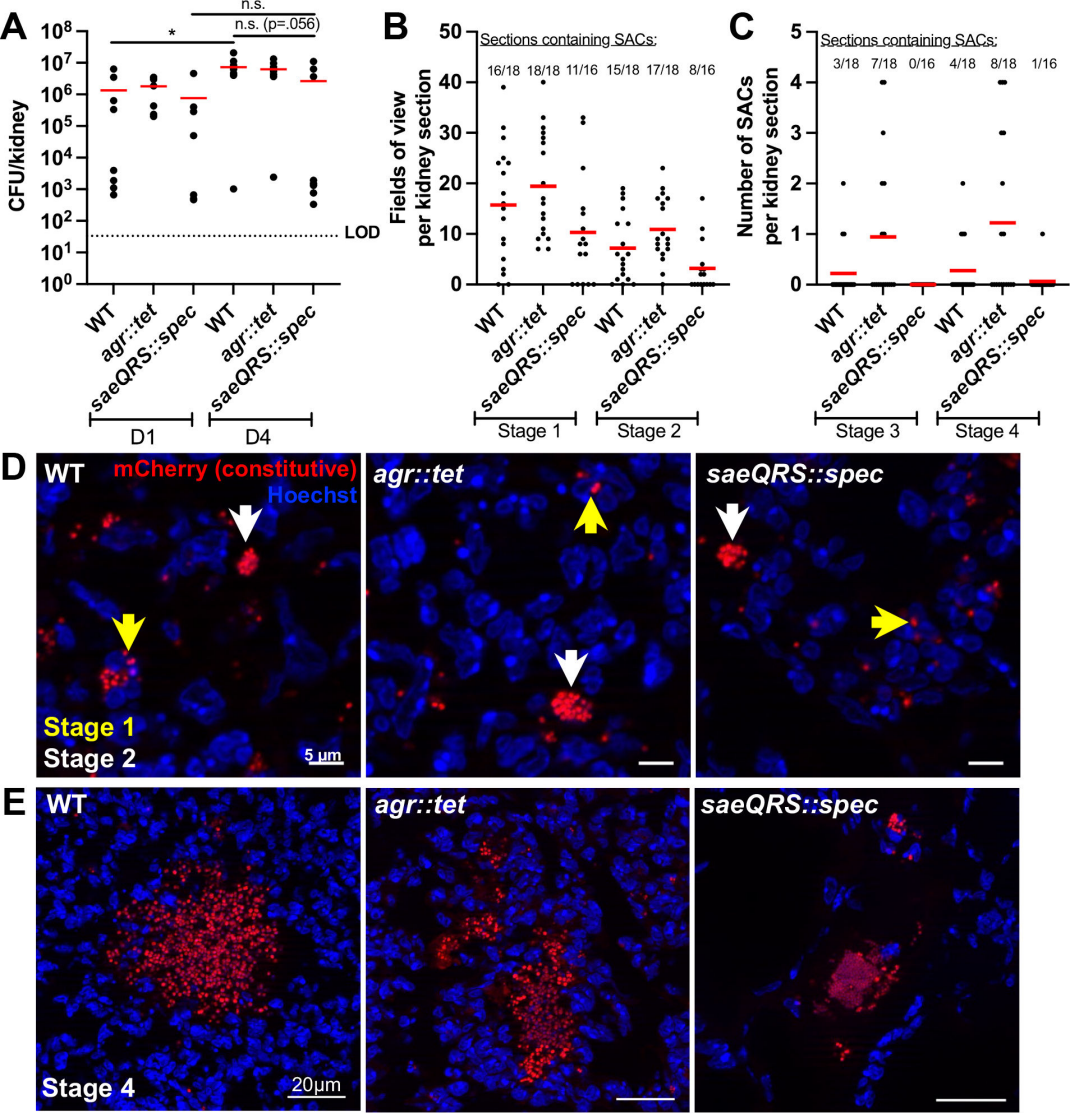
Loss of *sae* but not *agr* impacts abscess development. C57BL/6 mice were inoculated with WT, *agr::tet*, or *saeQRS::spec* strains expressing mCherry constitutively. Mice were sacrificed at days 1 or 4, and the kidneys were harvested. Left kidneys were homogenized to quantify bacterial load (CFU/kidney), and right kidneys were fixed and processed for fluorescence microscopy. (**A**) Bacterial load, each dot represents one mouse. *N* = 7 to 9 mice per time point. Red bars: mean. Dotted line denotes the LOD. (**B**) Number of fields of view per section that contained stage 1 or stage 2 SACs (D4 p.i.). *N* = 8 to 9 mice (two sections per mouse). (**C**) Number of stage 3 or stage 4 SACs per section (D4 p.i.). *N* = 8 to 9 mice (two sections per mouse). (**D**) Representative images for data shown in panel B. Yellow and white arrows represent stage 1 and stage 2 events, respectively. (**E**) Representative images for data shown in panel C. Statistics: (**A**) Kruskal-Wallis one-way ANOVA with Dunn’s post-test. *P < 0.05, n.s.: not significant.

Given our data that Agr is inactive and Sae is active during kidney abscess development, we hypothesized that loss of *sae*, but not *agr*, would hinder abscess development. To test this, we performed infections with WT, *agr::tet* (29), and *saeQRS::spec* (30) strains constitutively expressing mCherry. Day 1 time points were used to assess relative bacterial seeding in the kidney, and day 4 was used to represent the peak of infection. CFU quantification indicated that the WT bacterial load increased between day 1 and day 4 p.i., but *saeQRS::spec* CFUs did not increase (Fig. 7A). To investigate abscess development*,* we performed microscopic analysis at day 4 p.i., where bacteria in two distinct sections were imaged from each tissue and categorized into abscess stages. Intracellular *S. aureus* (stage 1) and small extracellular clusters (stage 2) were observed in mice infected with each strain (Fig. 7B and D). However, major differences were detected in the ability of the *saeQRS::spec* strain to progress to later-stage SACs. While WT and *agr::tet* strains formed intact (stage 3) and dispersed (stage 4) SACs, the *saeQRS::spec* strain failed to form stage 3 SACs and formed only a single, small stage 4 SAC with aberrant morphology (Fig. 7C and E). While we expected a more pronounced impact on *saeQRS::spec* kidney CFUs, these data align with published research and indicate later time points may be needed to see an impact on kidney CFUs (31). Our data suggest that an active Sae system is necessary for the formation of SACs and kidney abscess development, while Agr is dispensable.

## DISCUSSION

The Agr and Sae master regulatory systems control the expression of a large arsenal of *S. aureus* virulence factors, including those that contribute to kidney abscess development (9, 17). However, when they are active, and if activity is restricted to specific bacterial subpopulations, it has remained largely unknown. To address this, we generated a modified categorization to define the stages of kidney abscess formation (Fig. 2A). Our staging was very similar to Cheng et al. (25), but we focused on events in the kidney, omitting the initial bloodstream survival stage (9, 25). We also utilized the USA300 LAC strain, which, unlike strain Newman (15, 32), can regulate Sae. Somewhat surprisingly, we observed a great deal of similarity in abscess development compared to strain Newman (9), which may be linked to our observation that Sae is largely active during abscess development. A notable exception was observed at early stages, when intracellular *S. aureus* within neutrophils exhibited an ON/OFF Sae phenotype (Fig. 4B and C). Given *sae* expression is required for abscess progression (Fig. 7B and C), this could indicate that ON and OFF cells have different intracellular fates. Alternatively, this could indicate that cross-complementation occurs, where Sae activity in a subset of cells may be sufficient to promote the progression of the bacterial population as a whole.

*sae* expression increased within mature SACs, and we observed three distinct *sae* expression patterns (Fig. 6). The set with higher *sae* expression at the periphery was consistent with the model that immune cell proximity induces heightened Sae activity. SACs with heightened *sae* expression at the center were instead consistent with Behera et al., where higher expression of Sae-regulated *nuc* occurred at the SAC center (33). Nutrient depletion can relieve CodY repression of *sae* expression (16). Consistent with this, we observed an increase in *sae* reporter expression in the extended stationary phase of culture (Fig. S2E and G). Differential nutrient availability promotes heterogeneity in other bacterial abscess and granuloma models (34, 35), and it is possible that nutrient limitation occurs at the SAC center, triggering *sae* expression. It is also likely that individual abscesses have distinct nutritional microenvironments, given the *S. aureus* inter-abscess heterogeneity in metal availability and siderophore production (36, 37). We would predict that the heterogeneity we observed is a cumulative effect of these factors.

While we observed the importance of Sae for abscess development, we saw surprisingly little Agr activity and found that *agr* was not required for kidney abscess progression. A previous study by García-Betancur et al. showed that downregulation of Agr activity can be driven by Mg^2+^, and Mg^2+^-rich organs like kidneys and bones were found to harbor higher proportions of Agr-inactive *S. aureus* cells (38). Additionally, the macrophage apolipoprotein B receptor (*Apobr*) is known to inhibit the Agr system, and its presence has been detected at the SAC-host interface within kidney abscesses (39). However, using a 3D *in vitro* model of SACs, Guggenberger et al. showed that Agr is required for the dispersion of SACs at later stages (26). Given that dispersal was observed with the *agr* mutant strain, additional experiments would be needed to determine the contribution of Agr to SAC dispersal in the kidney.

A key limitation of these findings is the reliance on endpoint analyses. Longitudinal tracking of individual abscesses over time could yield even more novel spatiotemporal information regarding virulence factor expression and abscess biology. Use of a luciferase reporter with IVIS technology can enable real-time monitoring of gene expression during infection, but due to limitations in resolution, spatial gene expression information cannot yet be obtained (5). Two recent studies have utilized 3D tissue culture-based SAC models that could potentially enable spatiotemporal monitoring (26, 40). These systems also offer the ability to incorporate human components, such as fibrinogen, immune cells, or tissue-specific cells, to facilitate translation of findings to human infection. Data obtained from these diverse but complimentary model systems can bolster our understanding of *S. aureus* pathogenesis and guide strategies for better management of staphylococcal infections.

## MATERIALS AND METHODS

### Bacterial strains and growth conditions

*S. aureus* USA300 LAC wild type (41) and its derivatives were grown in tryptic soy broth (TSB), and *Escherichia coli* DH5α and IM08B in Luria broth (LB), all at 37°C with aeration. Antibiotics were used at the following concentrations: kanamycin (50 µg/mL), chloramphenicol (5 µg/mL), tetracycline (4 µg/mL), and spectinomycin (250 µg/mL) for *S. aureus*; erythromycin (500 µg/mL) and chloramphenicol (25 µg/mL) for *E. coli*.

### Construction of *S. aureus* fluorescent reporter strains

To generate constitutive mCherry^+^ strains, the *P_sarA_::sod*RBS fragment from pOS1-*P_sarA_-sod*RBS-*sgfp* (30) was fused to a *S. aureus* codon-optimized *mCherry* (IDT) and cloned into pJC1111 for chromosomal integration at the SaPI1 *attC* attachment site (20). The plasmid was isolated from *E. coli*, electroporated into *S. aureus* strain RN9011 (20), and recombinants were selected on 0.1 mM cadmium chloride, and the plasmid was transferred into strain LAC by phage transduction.

For GFP reporters, pIMAY, a temperature-sensitive plasmid with a tetracycline-inducible anti-*secY* counter selection marker, was used (42). For the GFP^−^ control strain, pINT-*gfp* was constructed by assembling in pIMAY: *sod*RBS-*sgfp*, *kanR* cassette, and flanking homology arms for genome integration at a neutral site between SAUSA300_RS05730 and RS05735 (21). To construct pINT-*P_agrB_::gfp* and pINT-*P_saeP_::gfp*, promoter regions upstream of *agrB* and *saeP* were cloned into pINT-*gfp*. Plasmids were assembled and isolated from *E. coli* IM08B, then transformed directly into *S. aureus* LAC strains (43). For strains containing both fluorescent constructs, the GFP construct was added first. Transformants were selected on chloramphenicol at 28°C, chromosomal integration was facilitated by growth at 37°C, and recombinants were selected on 1 µg/mL anhydrotetracycline, as described in reference 42. Additional reporter construction details are included in the Supplemental Information.

### *In vitro* reporter characterization

Overnight cultures (16 h) were diluted 1:100 into fresh TSB and incubated at 37°C with aeration. At each time point, samples were added to black-walled, clear-bottom, 96-well plates for absorbance (OD_600nm_) and fluorescence measurements (GFP: 480ex/520em, mCherry: 560ex/610em) using a Synergy microplate reader (Biotek Instruments). Colonies on tryptic soy agar were imaged using a Biotek Cytation 5 cell imaging multimode reader (Agilent). For flow cytometry, 100 µL of the culture at the indicated OD was pelleted and fixed in 4% paraformaldehyde (PFA) in PBS overnight at 4°C. Samples were analyzed on a BD FACSymphony A3 flow cytometer. Data were analyzed using FlowJo v10.10.0 software (BD Biosciences).

### Murine infection model

All animal experiments were approved by the Johns Hopkins University Institutional Animal Care and Use Committee (Protocol#: MO20H330, MO23H310). *S. aureus* frozen stocks in 10% glycerol (prepared after 2 h of growth from a 1:50 back-dilution of overnight cultures) were washed and diluted in sterile PBS to 10^6^ CFU in 100 µL for inocula. 6- to 8-week-old female C57BL/6 mice (Jackson Laboratories) were inoculated intravenously via the retro-orbital (under isoflurane anesthesia) or tail vein (with manual restraint) routes. At the indicated time points post-inoculation, mice were euthanized using a lethal dose of isoflurane, followed by cervical dislocation, and the kidneys were harvested. Left kidneys were homogenized for CFU enumeration, and the right kidneys were fixed in 4% PFA overnight at 4°C for histology (exception: Fig. 1B, left/right CFU comparison).

### Fluorescence microscopy of mouse tissues

After fixation, the right kidneys were frozen-embedded in O.C.T. compound (Tissue-Tek, VWR) and stored at −80°C. A total of 10 µm sections were cut using a cryostat microtome (Microm HM 505e) and mounted on charged microscope slides (HistoBond, VWR). Sections were thawed in PBS at room temperature (RT) and stained with Hoechst (1:10,000 dilution in PBS) for 15 min. To stain immune cells, thawed sections were permeabilized using ice-cold methanol for 2 min, blocked with 2% bovine serum albumin (BSA) in PBS at RT for 1 h, then incubated with either APC-conjugated rat anti-mouse Ly6G antibody (Invitrogen: 17-9668-80) or rat anti-mouse CD68 primary antibody (BioRad: MCA1957T) diluted in 2% BSA overnight at 4°C. For CD68 detection, sections were washed in PBS and incubated with donkey anti-rat Alexa Fluor 647-conjugated secondary antibody (Invitrogen: A48272TR) for 1 h at RT and stained with Hoechst. To differentiate intracellular and extracellular *S. aureus*, unpermeabilized tissue sections were stained overnight at 4°C with rabbit anti-*S*. *aureus* (Invitrogen: PA1-7246) and rat anti-mouse Ly6G (BD Biosciences: 551459) primary antibodies, followed by incubation with goat anti-rabbit AMCA-conjugated (Jackson: 111-155-14) and donkey anti-rat Alexa Fluor 647-conjugated secondary antibodies for 1 h at RT. For the detection of total *S. aureus*, the same staining procedure was performed after methanol permeabilization. Coverslips were mounted with ProLong Gold (Invitrogen). Two to three sections per mouse were imaged using a Zeiss Axio Observer 7 inverted fluorescent microscope with a 63× oil objective and Apotome.2. Images were captured with an Axiocam 702 mono camera (Zeiss) and processed using ZEN3.10 software.

### GFP stability

Mid-log phase *S. aureus* harboring the pCM29 plasmid containing *P_sarAP1_::sgfp* (44) was pelleted and resuspended in 200 µL of PBS + kanamycin (50 µg/mL) to inhibit protein translation. Samples were added to black-walled, clear-bottom, 96-well plates and incubated at 37°C in a Synergy microplate reader (Biotek Instruments). OD and GFP fluorescence measurements were taken every 15 min.

### Quantitative reverse transcription PCR to detect bacterial transcripts

Bacteria were grown for the indicated times, pelleted, and fixed in 4% PFA at 4°C overnight. After three PBS washes, cells were resuspended in 100 mM Tris-HCl, 1 mg/mL lysostaphin, and 0.5 µL RNaseOUT (Invitrogen) and incubated for 30 min at 37°C. RNA was isolated using the RNeasy FFPE kit (Qiagen), and gDNA was eliminated using the TURBO DNA-free kit (Invitrogen). cDNA was synthesized using random primers and the ProtoScript II First Strand cDNA Synthesis Kit (New England Biolabs). Quantitative PCR (qPCR) was performed with PowerUp SYBR Green Master Mix (Applied Biosystems) using primers for *agrB*, *saeP*, *gfp,* and *16s* on a QuantStudio 6 Pro system (Applied Biosystems). Relative quantification was performed using the ∆∆C_T_ method relative to the earlier time point. All kits and reagents were used according to the manufacturers’ protocols.

### Image analysis

#### Abscess stage criteria

Stage 1: intracellular *S. aureus* (single cells or clusters) in contact with host cell nuclei (Hoechst-stained).

Stage 2: small extracellular clusters (distanced from host cells).

Stage 3: large compact or loosely packed SACs, surrounded by a continuous fibrin layer, with no direct host cell contact.

Stage 4: dispersed SAC with a discontinuous fibrin layer and host cells contacting *S. aureus* at multiple points.

Stage 1 and 2 events were excluded if found in the same field as stage 3 or 4 SACs, to ensure these events represented early stages preceding SAC formation, and not ruptured SACs.

#### Temporal expression pattern

Volocity image analysis software was used to quantify bacterial area, mCherry, and GFP signals. Objects (individual bacterial cells/cluster/SACs) were defined by mCherry signal, and area, sum mCherry, and sum GFP fluorescence were measured. Sum GFP/sum mCherry ratios were calculated. Objects <0.4 µm^2^ (artifacts) were excluded, but faint mCherry^+^ cells were included. For stage 1, at least 100 events or events from two fields per section (whichever was greater) were analyzed. For stages 2–4, all events per section were analyzed to avoid selection bias. If <5 events were present, an additional section (≥150 µm apart) was analyzed to reach five events per mouse.

#### Spatial expression pattern

For stage 3 SACs, one central region of interest (ROI: 4 × 4 µm) and eight peripheral ROIs were selected. For stage 4 SACs, one interior ROI (away from host cells) and eight ROIs along rupture sites (in direct contact with host cells) were selected. Within these ROIs, objects were detected as described above, and the GFP/mCherry ratio for each ROI was calculated using the sum GFP and sum mCherry values. The GFP/mCherry ratio of the center and the average GFP/mCherry value of the eight peripheral (or in direct contact) ROIs are shown.

### Comparison of abscess stage distribution in WT, *agr*::tet, and *saeQRS*::spec infections

Two sections approximately 300 µm apart were analyzed per mouse. All bacteria-containing fields were imaged as described above. Due to the high abundance of stages 1 and 2, the number of fields containing at least one stage 1 or 2 event was counted per section. For stages 3 and 4, the numbers of SACs per section were quantified.

### Statistical analysis

GraphPad Prism 10 was used to graph all data and for statistical analyses. The statistical tests used are described in the respective figure legends. A *P *value of <0.05 was considered significant.

## SUPPLEMENTAL MATERIAL

Supplemental Figures mbio.02043-25-s0001.docx Figures S1 to S4.

Supplemental Data File mbio.02043-25-s0002.xlsx All raw data.

Supplemental Material mbio.02043-25-s0003.docx Supplemental legends and additional methods.

Table S1 mbio.02043-25-s0004.docx Sample size information for microscopy analyses.

ASM does not own the copyrights to Supplemental Material that may be linked to, or accessed through, an article. The authors have granted ASM a non-exclusive, world-wide license to publish the Supplemental Material files. Please contact the corresponding author directly for reuse.
