# Supplementary material for: Staphylococcus aureus exhibits spatiotemporal heterogeneity in Sae activity during kidney abscess development
Source: mBio. 2025 Nov 13;16(12):e02043-25. doi: 10.1128/mbio.02043-25 (PMC12691657; doi:10.1128/mbio.02043-25)
Supplement: Supplemental Figures — Figures S1 to S4. [file mbio.02043-25-s0001.docx]

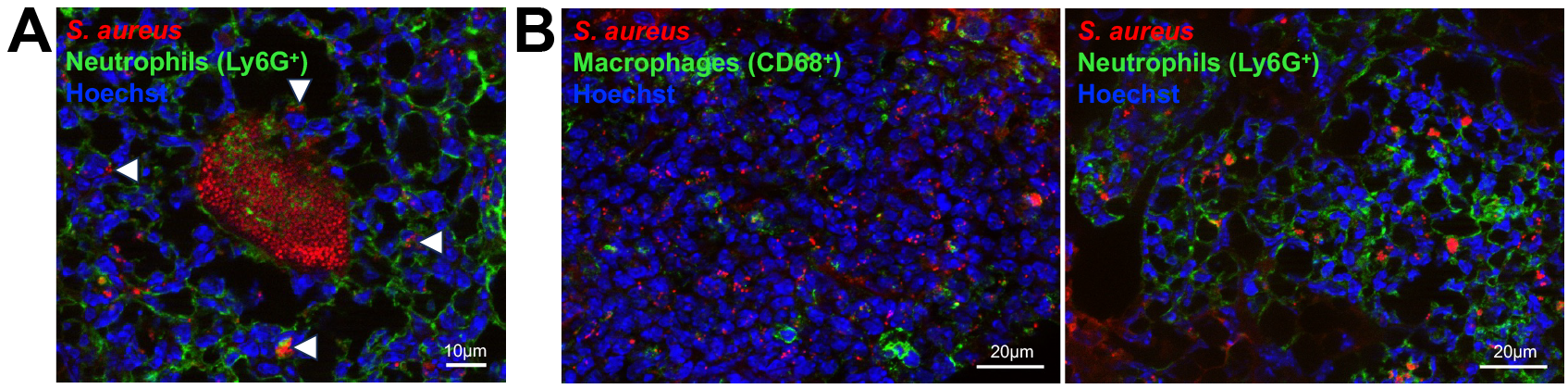


**Supplemental Figure 1: Immune cell localization in mouse kidney abscesses.** C57BL/6 mice were inoculated with the *S. aureus* GFP^-^ control strain. Mice were sacrificed at day 4 and kidneys were harvested and processed for fluorescence microscopy. A) Representative image showing localization of neutrophils (Ly6G^+^) around a dispersed stage 4 SAC. White arrows: *S. aureus* interacting with neutrophils in the vicinity of a dispersed SAC. B) Representative image showing localization of macrophages (CD68^+^, left panel) and neutrophils (Ly6G^+^, right panel) around stage 1 and stage 2 events.


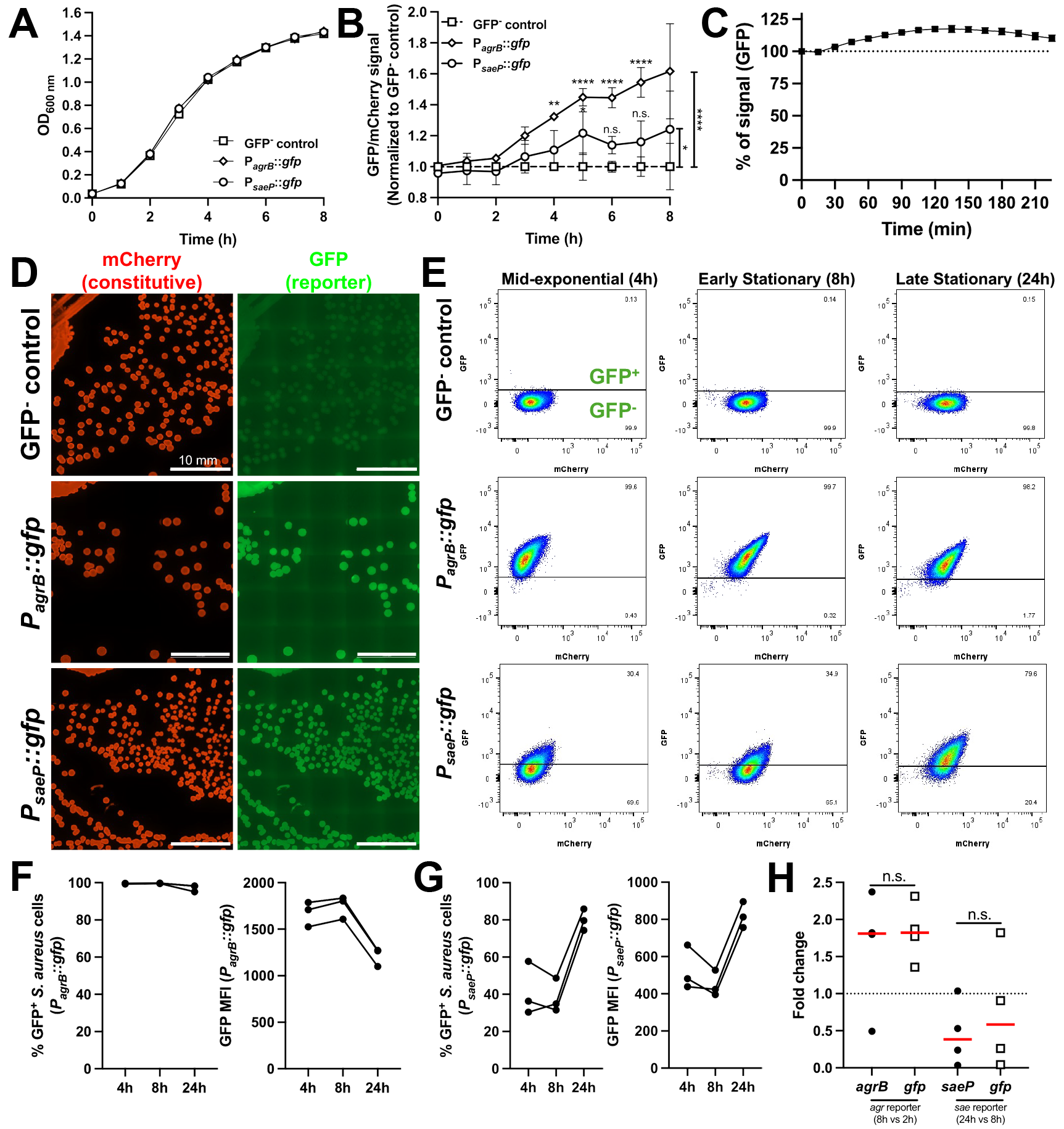


**Supplemental Figure 2: Characterizing *S. aureus* fluorescent reporter strains during growth *in vitro* in tryptic soy broth or agar.** Overnight cultures of *S. aureus* were diluted 1:100 in fresh TSB and incubated at 37˚C with shaking. At the indicated timepoints, A) absorbance (OD_600nm_), B) GFP, and mCherry fluorescence were measured using a microplate reader. Mean ± SD of three biological replicates are shown. Black dotted line (baseline): average GFP/mCherry value of the GFP^-^ control at the indicated timepoints. C) GFP stability. % of GFP signal in mid-log phase *S. aureus* expressing constitutive GFP (44), % is relative to the timepoint where kanamycin was added (0h) to inhibit protein translation. D) Colonies of *S. aureus* on tryptic soy/kanamycin agar imaged after overnight growth at 37˚C. E) Representative flow cytometry plots showing reporter expression in GFP^-^ control, *agr*, and *sae* reporter strains at the indicated growth phases and timepoints. F, G) Percentage of GFP^+^ and mean fluorescence intensity (MFI) of GFP in *agr* (F) or *sae* (G) reporter cells at the indicated timepoints, measured by flow cytometry. Mean values represent 50,000 cells per replicate, N = 3 biological replicates. Lines connect values from the same replicate. H) qRT-PCR detection of either *agrB* or *saeP* alongside *gfp* in the indicated reporter strains. Fold change in transcript levels is shown relative to the indicated timepoints. Statistics: B) Two-way ANOVA with Tukey’s test, comparison to GFP^-^ control; H) Kruskal-Wallis one-way ANOVA with Dunn’s post-test. *****p*<0.0001, ***p*<0.01, **p*<0.05, n.s.: not significant.


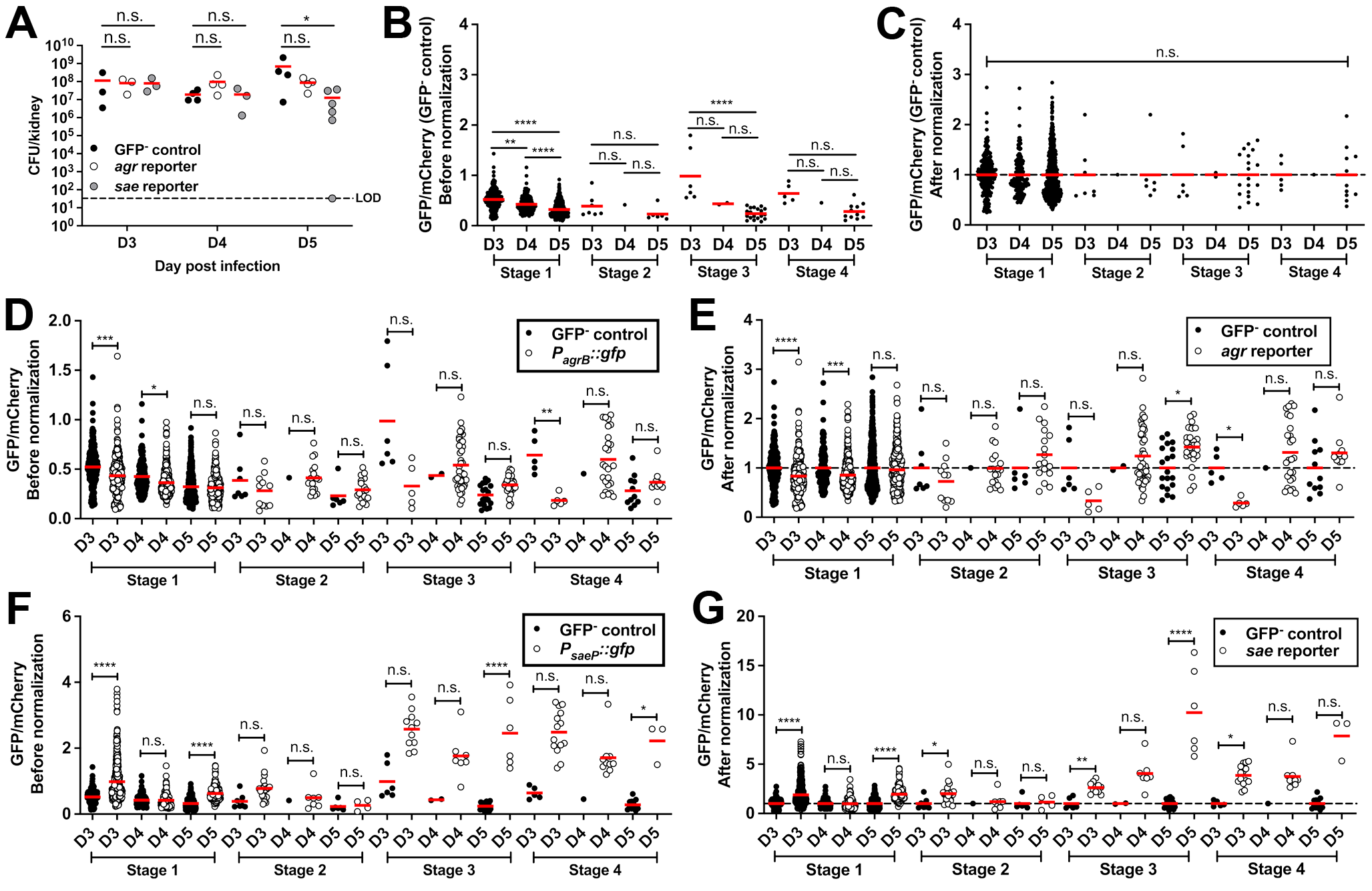


**Supplemental Figure 3: Reporter expression in mouse kidney abscesses.** C57BL/6 mice were inoculated with GFP^-^ control, *agr*, or *sae* reporter strains. Mice were sacrificed at days 3, 4, or 5 (D3, D4, D5) and kidneys were harvested. Left kidneys were homogenized to quantify bacterial load (CFU/kidney) and right kidneys were fixed and processed for fluorescence microscopy. A) CFU/kidney of mice infected with *S. aureus* reporter strains at the indicated timepoint. Each dot represents one mouse. N = 3 to 6 mice. B) GFP/mCherry ratio of GFP^-^ control abscesses (stages 1 to 4) at the indicated timepoints. C) GFP/mCherry ratio of individual GFP^-^ control events after normalization to the average value of day- and stage-matched GFP^-^ control events (represented by the black dashed baseline at Y=1 in panel C, E and G). D - G) Comparison in reporter expression of *agr* or *sae* reporter events (stages 1 to 4) to day- and stage-matched GFP^-^ control events; GFP/mCherry ratios without normalization (panels D and F) and after normalization to the GFP^-^ control (panels E and G) are shown. Dots represent intracellular *S. aureus* (single/cluster, stage 1), individual extracellular clusters (stage 2) or SACs (stages 3 and 4) in panels B to G. N = 3 to 5 mice per timepoint. Red bars represent mean. Statistics: A), B), C), E) and G) Kruskal-Wallis one-way ANOVA with Dunn’s test. *****p*<0.0001, ****p*<0.001, ***p*<0.01, **p*<0.05, n.s.: not significant.


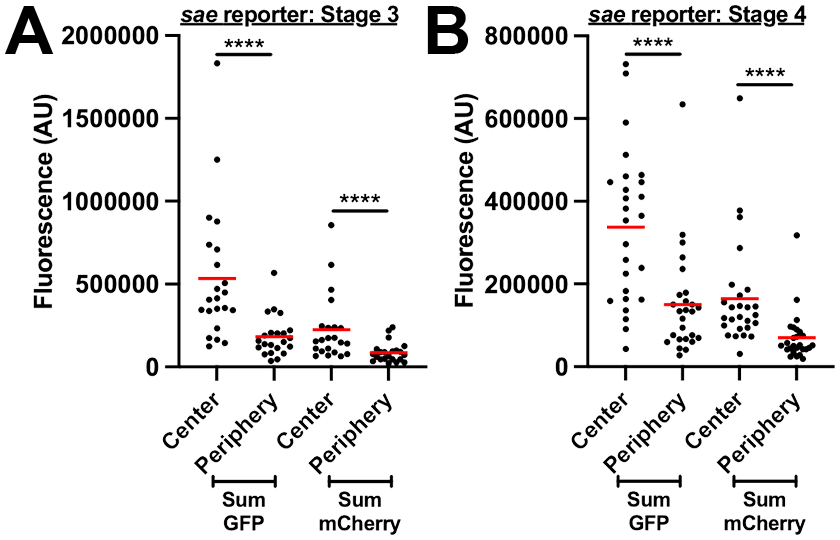


**Supplemental Figure 4: GFP (reporter) and mCherry (constitutive) signals within individual SACs.** C57BL/6 mice were inoculated with the *sae* reporter strain. Mice were sacrificed at days 3, 4, or 5, and kidneys were harvested and processed for fluorescence microscopy. Stage 3 (panel A): one region of interest (ROI, 4µm^2^) at the center and 8 ROIs along the periphery were selected. The 8 peripheral ROI values were averaged. Stage 4 (panel B): one ROI in the center (away from host cells) and 8 ROIs along the rupture (periphery, in contact with host cells) were selected. The peripheral ROI values were averaged. Shown are sum GFP and sum mCherry values at the center and periphery of A) stage 3, combined data from D3 to D5 (GFP/mCherry shown in Fig 6C); and B) stage 4, combined data from D3 to D5 (GFP/mCherry shown in Fig 6E). N = 3 to 5 mice per timepoint. Statistics: Wilcoxon matched-pairs test. *****p*<0.0001.
