# Supplementary material for: Staphylococcus aureus exhibits spatiotemporal heterogeneity in Sae activity during kidney abscess development
Source: mBio. 2025 Nov 13;16(12):e02043-25. doi: 10.1128/mbio.02043-25 (PMC12691657; doi:10.1128/mbio.02043-25)
Supplement: Supplemental Material — Supplemental legends and additional methods. [file mbio.02043-25-s0003.docx]

**Supplemental Information: ‘***Staphylococcus aureus* exhibits spatiotemporal heterogeneity in Agr and Sae activity during kidney abscess development’

**Supporting Information: Figure and Table Legends**

**Supplemental Figure 1: Immune cell localization in mouse kidney abscesses.** C57BL/6 mice were inoculated with the *S. aureus* GFP^-^ control strain. Mice were sacrificed at day 4 and kidneys were harvested and processed for fluorescence microscopy. A) Representative image showing localization of neutrophils (Ly6G^+^) around a dispersed stage 4 SAC. White arrows: *S. aureus* interacting with neutrophils in the vicinity of a dispersed SAC. B) Representative image showing localization of macrophages (CD68^+^, left panel) and neutrophils (Ly6G^+^, right panel) around stage 1 and stage 2 events.

**Supplemental Figure 2: Characterizing *S. aureus* fluorescent reporter strains during growth *in vitro* in tryptic soy broth or agar.** Overnight cultures of *S. aureus* were diluted 1:100 in fresh TSB and incubated at 37˚C with shaking. At the indicated timepoints, A) absorbance (OD_600nm_), B) GFP, and mCherry fluorescence were measured using a microplate reader. Mean ± SD of three biological replicates are shown. Black dotted line (baseline): average GFP/mCherry value of the GFP^-^ control at the indicated timepoints. C) GFP stability. % of GFP signal in mid-log phase *S. aureus* expressing constitutive GFP (44), % is relative to the timepoint where kanamycin was added (0h) to inhibit protein translation. D) Colonies of *S. aureus* on tryptic soy/kanamycin agar imaged after overnight growth at 37˚C. E) Representative flow cytometry plots showing reporter expression in GFP^-^ control, *agr*, and *sae* reporter strains at the indicated growth phases and timepoints. F, G) Percentage of GFP^+^ and mean fluorescence intensity (MFI) of GFP in *agr* (F) or *sae* (G) reporter cells at the indicated timepoints, measured by flow cytometry. Mean values represent 50,000 cells per replicate, N = 3 biological replicates. Lines connect values from the same replicate. H) qRT-PCR detection of either *agrB* or *saeP* alongside *gfp* in the indicated reporter strains. Fold change in transcript levels is shown relative to the indicated timepoints. Statistics: B) Two-way ANOVA with Tukey’s test, comparison to GFP^-^ control; H) Kruskal-Wallis one-way ANOVA with Dunn’s post-test. *****p*<0.0001, ***p*<0.01, **p*<0.05, n.s.: not significant.

**Supplemental Figure 3: Reporter expression in mouse kidney abscesses.** C57BL/6 mice were inoculated with GFP^-^ control, *agr*, or *sae* reporter strains. Mice were sacrificed at days 3, 4, or 5 (D3, D4, D5) and kidneys were harvested. Left kidneys were homogenized to quantify bacterial load (CFU/kidney) and right kidneys were fixed and processed for fluorescence microscopy. A) CFU/kidney of mice infected with *S. aureus* reporter strains at the indicated timepoint. Each dot represents one mouse. N = 3 to 6 mice. B) GFP/mCherry ratio of GFP^-^ control abscesses (stages 1 to 4) at the indicated timepoints. C) GFP/mCherry ratio of individual GFP^-^ control events after normalization to the average value of day- and stage-matched GFP^-^ control events (represented by the black dashed baseline at Y=1 in panel C, E and G). D - G) Comparison in reporter expression of *agr* or *sae* reporter events (stages 1 to 4) to day- and stage-matched GFP^-^ control events; GFP/mCherry ratios without normalization (panels D and F) and after normalization to the GFP^-^ control (panels E and G) are shown. Dots represent intracellular *S. aureus* (single/cluster, stage 1), individual extracellular clusters (stage 2) or SACs (stages 3 and 4) in panels B to G. N = 3 to 5 mice per timepoint. Red bars represent mean. Statistics: A), B), C), E) and G) Kruskal-Wallis one-way ANOVA with Dunn’s test. *****p*<0.0001, ****p*<0.001, ***p*<0.01, **p*<0.05, n.s.: not significant.

**Supplemental Figure 4: GFP (reporter) and mCherry (constitutive) signals within individual SACs.** C57BL/6 mice were inoculated with the *sae* reporter strain. Mice were sacrificed at days 3, 4, or 5, and kidneys were harvested and processed for fluorescence microscopy. Stage 3 (panel A): one region of interest (ROI, 4µm^2^) at the center and 8 ROIs along the periphery were selected. The 8 peripheral ROI values were averaged. Stage 4 (panel B): one ROI in the center (away from host cells) and 8 ROIs along the rupture (periphery, in contact with host cells) were selected. The peripheral ROI values were averaged. Shown are sum GFP and sum mCherry values at the center and periphery of A) stage 3, combined data from D3 to D5 (GFP/mCherry shown in Fig 6C); and B) stage 4, combined data from D3 to D5 (GFP/mCherry shown in Fig 6E). N = 3 to 5 mice per timepoint. Statistics: Wilcoxon matched-pairs test. *****p*<0.0001.

**Supplemental Table 1: Sample size information for microscopy analyses.** C57BL/6 mice were inoculated with *S. aureus* GFP^-^ control, *agr*, or *sae* reporter strains. At the indicated timepoints, mice were sacrificed, and kidneys were harvested. Left kidneys were used for CFU enumeration, and right kidneys were processed for microscopic examination. N represents the number of mice. 2-3 sections per mice were imaged and analyzed. The number of events analyzed for each mouse are shown.

**Supplemental Data File.** All raw data from the manuscript is provided in this file.

**Supplemental Information File:** File includes a pINT-*gfp* plasmid map, *agrB* and *saeP* promoter sequences included in fluorescent reporter constructs, and additional methodological details for *S. aureus* fluorescent reporter strain construction.

**pINT-*gfp* plasmid map**


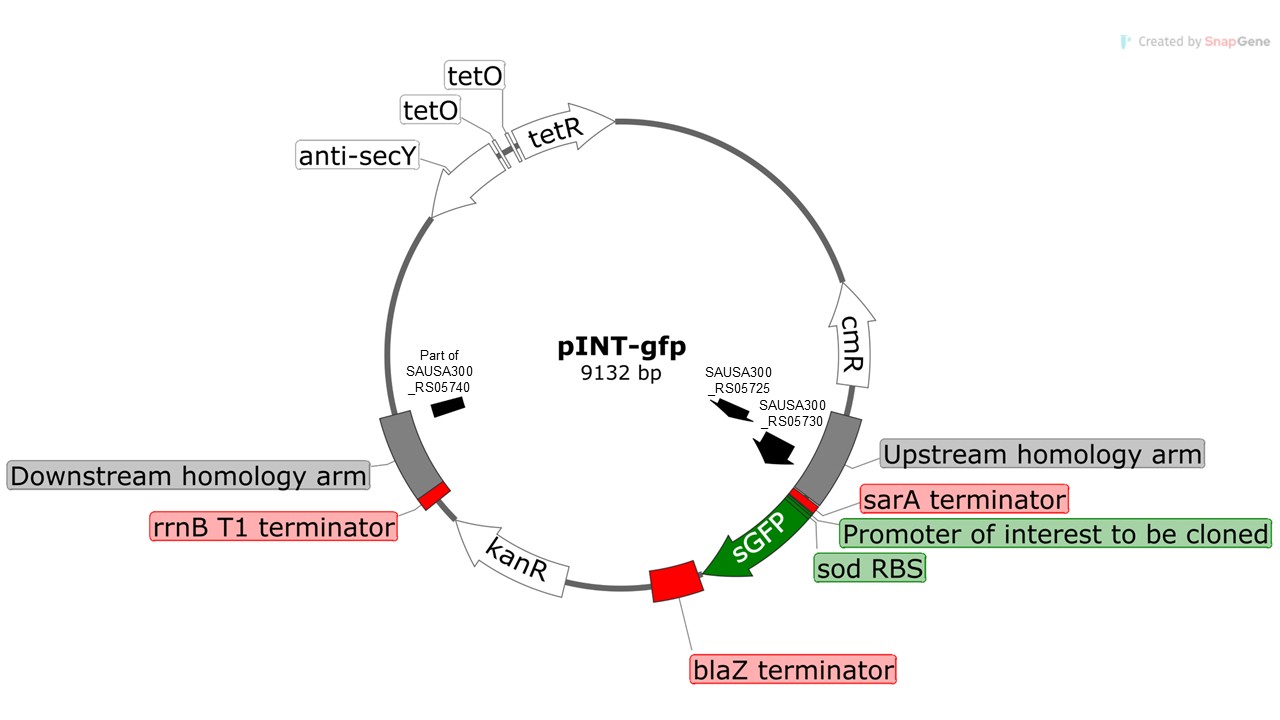


**Plasmid map of pINT-*gfp* showing the following features:** a tetracycline-inducible anti-*secY* sequence, tetracycline operator (*tetO*) and tetracycline repressor (*tetR*) sequences, and a chloramphenicol resistance module (c*mR*), all originally present in pIMAY. A single large fragment (synthesized by Genewiz) containing the *sod* RBS-*sgfp* fragment, a kanamycin resistance module (kanR), the *sarA*, *blaZ*, and *rrnB* T1 terminators, and flanking homology arms was cloned into pIMAY by Gibson assembly. The upstream homology arm includes the *SAUSA300_RS05725* and *RS05730* ORFs, while the downstream homology arm consists of a portion of the *RS05740* ORF. Map generated using SnapGene® software (from Dotmatics; available at snapgene.com)

***agrB* promoter sequence**

GCATTTATTTTCCAATTTTTCTTAACTAGTCGTTTTTTATTCTTAACTGTAATTTTTTTTATGTTAAAATATTAAATACAAATTACATTTAACAGTTAAGTATTTATTTCCTACAGTTAGGCAATATAATGATAAAAGATTGTACTAAATCGTATAATGACAGTG

***saeP* promoter sequence**

GTGGCAAAAGGTTTATAAATTTTAATACCAAAACTATTAAACACTTCTGATATTCTTAGTTCAAAATATCAGAAGTGTTTTATAGTGTTATCTAGTTCAGATAAATATTTCCTTACTTAAAAAAACGCCCTCCTCTTATTTTGACCCCTATTTATTTAAATCAGACAATTATTTTCATTTTCAAATTATTCTTTCTTCAATATTAGTTAAGCGATATTTAAACGAAGTTAAGAATTAGTTAATGGCATATTATTTGCCTTCATTTTAAACTTAACTTATCAAATTG

*Construction of S. aureus fluorescent reporter strains.* To generate constitutive *mCherry*^+^ strains, a *P_sarA_*::*sod* RBS::*mCherry* (*sarA* promoter and *sod* ribosomal binding site fused upstream of mCherry) cassette was cloned into pJC1111 for chromosomal integration at the SaPI1 *attC* attachment site (1). The *P_sarA_*::*sod* RBS fragment amplified from pOS1-*P_sarA_*-*sod*RBS-*sgfp* (2), and the codon-optimized *mCherry* ORF ((3); synthesized by IDT) were inserted into pJC1111 by Gibson assembly. The resulting plasmid was isolated from *E. coli* transformants and electroporated into RN9011 (1), and recombinants were selected on 0.1 mM cadmium chloride. The integrated cassette was then moved to LAC by transduction. To generate *mCherry*^+^ *agr* and *sae* mutant strains, the pJC1111-*P_sarA_::sod*RBS*::mCherry*  plasmid was transduced into LAC *agr::tet* (4) and *saeQRS::spec* strains (2, 5), with selection as above.

For the GFP reporter constructs, pIMAY, a temperature sensitive plasmid with a tetracycline-inducible anti-*secY* counter selection marker, was used (6). For generation of the GFP^-^ control strain, pINT-*gfp* was constructed by cloning a single large fragment into pIMAY by Gibson assembly. This fragment contained the following components: the *S. aureus* *sarA* terminator, *sod* RBS-*sgfp* fragment, the *blaZ* terminator (from pJC1111), a *kanR* module (from pBT-*kanR*), the *E. coli* *rrnB* T1 terminator, and flanking homology arms for genome integration at a neutral site between *SAUSA300_RS05730* and *RS05740* (based on the USA300_FPR3757 NCBI RefSeq: NC_007793.1) (7).

To construct pINT-*P_agrB_*::*gfp* and pINT-*P_saeP_*::*gfp*, promoter regions upstream of *agrB* and *saeP* were cloned into pINT-*gfp*. The resulting plasmids were transformed into *E. coli* IM08B and then into LAC, as described in (8). Transformants were selected on chloramphenicol at 28˚C. Chromosomal integration was facilitated by growth at 37˚C, and double cross over recombinants were selected on 1 µg/ml anhydrotetracycline, as described in (6). Finally, the pJC1111-*P_sarA_::sod* RBS*::mCherry*  plasmid was transduced into the *gfp*^+^ strains to generate *mCherry*^+^ *gfp*^+^ strains, with selection as above.

**Supplemental Information References**

1. Chen J, Yoong P, Ram G, Torres V, Novick R. Single-copy vectors for integration at the SaPI1 attachment site for *Staphylococcus aureus*. Plasmid. 2014;76:1-7.

2. Benson M, Lilo S, Nygaard T, Voyich J, Torres V. Rot and SaeRS cooperate to activate expression of the staphylococcal superantigen-like exoproteins. J Bacteriol. 2012;194(16):4355-65.

3. Kato F, Nakamura M, Sugai M. The development of fluorescent protein tracing vectors for multicolor imaging of clinically isolated *Staphylococcus aureus*. Sci Rep. 2017;7(1):2865.

4. Benson M, Lilo S, Wasserman G, Thoendel M, Smith A, Horswill A, et al. *Staphylococcus aureus* regulates the expression and production of the staphylococcal superantigen-like secreted proteins in a Rot-dependent manner. Mol Microbiol. 2011;81(3):659-75.

5. DuMont A, Yoong P, Surewaard B, Benson M, Nijland R, van Strijp J, et al. *Staphylococcus aureus* elaborates leukocidin AB to mediate escape from within human neutrophils. Infect Immun. 2013;81(5):1830-41.

6. Monk I, Shah I, Xu M, Tan M, Foster T. Transforming the untransformable: application of direct transformation to manipulate genetically *Staphylococcus aureus* and *Staphylococcus epidermis*. MBio. 2012;3(2):e00277-11.

7. Plaut R, Mocca C, Prabhakara R, Merkel T, Stibitz S. Stably luminescent *Staphylococcus aureus* clinical strains for use in bioluminescent imaging. PLoS One. 2013;8(3).

8. Monk I, Tree J, Howden B, Stinear T, Foster T. Complete bypass of restriction systems for major *Staphylococcus aureus* lineages. mBio. 2015;6(3):e00308-15.
