# Supplementary material for: Staphylococcus aureus exhibits spatiotemporal heterogeneity in Sae activity during kidney abscess development
Source: mBio. 2025 Nov 13;16(12):e02043-25. doi: 10.1128/mbio.02043-25 (PMC12691657; doi:10.1128/mbio.02043-25)
Supplement: Table S1 — Sample size information for microscopy analyses. [file mbio.02043-25-s0004.docx]

|  |  | **GFP^-^ control** | ***P_agrB_::gfp*** | ***P_saeP_::gfp*** |
| --- | --- | --- | --- | --- |
| Day 3 | Stage 1 | 111, 0, 105 (N=3) | 116, 31, 101 (N=3) | 179, 169, 122 (N=3) |
|  | Stage 2 | 3, 0, 4 (N=3) | 3, 5, 3 (N=3) | 4, 7, 9 (N=3) |
|  | Stage 3 | 0, 4, 2 (N=3) | 2, 3, 0 (N=3) | 5, 1, 5 (N=3) |
|  | Stage 4 | 0, 1, 4 (N=3) | 0, 5, 0 (N=3) | 2, 1, 11 (N=3) |
| Day 4 | Stage 1 | 39, 71, 32, 4 (N=4) | 110, 202, 242 (N=3) | 105, 0, 61 (N=3) |
|  | Stage 2 | 1, 0, 0, 0 (N=4) | 6, 5, 7 (N=3) | 7, 0, 0 (N=3) |
|  | Stage 3 | 0, 0, 0, 2 (N=4) | 19, 11, 12 (N=3) | 3, 1, 3 (N=3) |
|  | Stage 4 | 0, 0, 0, 1 (N=4) | 12, 7, 6 (N=3) | 5, 1, 4 (N=3) |
| Day 5 | Stage 1 | 259, 124, 131 (N=3) | 108, 111, 129 (N=3) | 124, 0, 81 (N=3) |
|  | Stage 2 | 3, 0, 3 (N=3) | 4, 8, 0, 5 (N=4) | 0, 0, 1, 2, 1 (N=5) |
|  | Stage 3 | 12, 0, 0, 7 (N=4) | 2, 9, 15 (N=3) | 1, 0, 0, 4, 2 (N=5) |
|  | Stage 4 | 6, 0, 0, 5 (N=4) | 0, 5, 5 (N=3) | 1, 0, 0, 1, 1 (N=5) |
